# Tick-Borne Rickettsiosis in Traveler Returning from Honduras

**DOI:** 10.3201/eid1508.090172

**Published:** 2009-08

**Authors:** Lin H. Chen, Mary E. Wilson

**Affiliations:** Mount Auburn Hospital, Cambridge, Massachusetts, USA (L.H. Chen, M.E. Wilson); Harvard Medical School, Boston, Massachusetts, USA (L.H. Chen, M.E. Wilson); Harvard School of Public Health, Boston (M.E. Wilson)

**Keywords:** Rickettsiosis, tick-borne disease, tick typhus, vector-borne infections, disease in traveler, Honduras, letter

**To the Editor:** Although tick-borne rickettsioses are widespread globally, few reports document their presence in Central America (*1*). Serosurveys detected rickettsial antibodies in humans in Central America in 1971 in Costa Rica, Honduras, Nicaragua, and Panama (*2*,*3*). An outbreak of rickettsial illness was reported to have occurred in Costa Rica in 1974, where 2 case clusters affected 6 of 15 family members (*4*). A rickettsial organism was isolated from a patient who died in Panama in 1950 (*5*), and more recently *Rickettsia rickettsii* was confirmed in a fatal case in Panama (*6*). We report a patient with serologic evidence of rickettsiosis after a tick bite sustained during travel in Honduras.

A 51-year-old man sought medical evaluation after returning from travel to Roatan, Honduras, where he was bitten by a tick in the lower abdomen. He reported erythema and induration at the site of the tick bite with associated central necrosis. He described an illness with headache, fever, weakness, dizziness, abdominal discomfort, diarrhea, flu-like symptoms, and respiratory symptoms affecting him 1–2 weeks after the tick bite. He was evaluated while still traveling and received multiple diagnoses, including malaria, respiratory infection, and parasites, and was given chloroquine, primaquine, penicillin, and mebendazole. His condition improved. He returned to the United States 2 months after the tick bite.

His travel history included Thailand, Jamaica, Aruba, the Bahamas, Belize, Germany, Spain, Hungary, the Netherlands, and New Zealand. The patient recalled removing ticks from his body as a child in Maryland but had no associated illness. He denied any recent tick bite other than the one in Honduras, where he had close contact with a horse and dogs and was frequently outdoors.

Results of his physical examination were unremarkable; routine laboratory studies showed values within reference ranges. No antibodies to *Borrelia burgdorferi* or *Plasmodium* spp. were detected.

Serologic analysis for rickettsia, performed by a commercial laboratory (Focus Diagnostics, Cypress, CA, USA) and the Centers for Disease Control and Prevention (Atlanta, GA, USA), showed elevated titers (Table). The patient took doxycycline (100 mg 2×/d) for 10 days and subjectively improved.

**Table:** *Rickettsia* spp. serologic titers for a man who returned from Honduras, 2005*

Test	Titers on specific date				
	May 26	Jun 10	Jul 14	Aug 25	Nov 22
RMSF IgG†	Positive, >1,024		Positive, >1,024	Positive, >1,024	Positive, >1,024
RMSF IgM†	Positive, 512		Positive, 256	Positive, 128	Positive, 256
*R. conorii* IgG†			>1,024	512	512
*R*. *conorii* IgM†			64	64	64
*R. africae* IgG‡		2,048		4,096	
*R. africae* IgM‡		512			
*R. conorii* IgG‡		2,048		4,096	
*R. conorii* IgM‡		512			
*R. rickettsii* IgG‡		1,024			
*R. rickettsii* IgM‡		256			

*Reference titer for negative/normal result is <64. RMSF, Rocky Mountain spotted fever; Ig, immunoglobulin. †Testing done by Focus Diagnostics, Cypress, CA, USA. ‡Testing done by Centers for Disease Control and Prevention, Atlanta, GA, USA.

On the basis of infections documented in the Americas, *R. rickettsii*, *R. africae*, and other less well-known rickettsial pathogens such as *R. parkeri* or *R. massiliae* (*1*) are possible etiologic agents in our case. The most common human-biting tick in Central America is *Amblyomma cajennense*, which is a known vector of *R. rickettsii*. Serosurveys have suggested the presence of *R. rickettsii* infection in the Yucatan (*7*), and PCR has confirmed a case of *R. rickettsii* in that region (*8*). *Rhipicephalus sanguineus,* the vector of *R. conorii* (boutonneuse fever) in the Mediterranean, is also found in Mexico, but to date no transmission of *R. conorii* has been documented in the Americas. *R. sanguineus* was implicated in a cluster of human Rocky Mountain spotted fever cases in the United States in 2002–2004 (*9*).

Our patient had not traveled to the areas of the Caribbean where transmission of *R. africae* has been documented, but he had lived in areas of the United States with potential transmission of rickettsial infections. *R. parkeri* is a newly recognized pathogen in the Americas (*10*). Its vectors (*A. maculatum* and *A. triste*) are found in parts of North, Central, and South America. The illness caused by *R. parkeri* appears to be less severe than Rocky Mountain spotted fever and could be consistent with our patient’s illness. Because antibodies against *R. parkeri* and *R. rickettsii* cross-react, serologic analysis is of little use for differentiating these 2 organisms.

The history and serologic findings for our patient suggest a recent tick-borne rickettsiosis, most likely acquired in Honduras. However, we can neither confirm that infection was recent nor confirm the species. The history of a tick bite and description of the skin lesion are consistent with an eschar, but no physical evidence of the tick or eschar remained at the time of evaluation. Diagnosis of rickettsial infection can be confirmed by demonstrating at least a 4-fold increase in titers between acute-phase and convalescent-phase serum samples, by identification of rickettsiae in an acute-phase serum or tissue sample, or by culture. Use of an immunofluorescent antibody assay alone does not identify the specific agent causing spotted fever.

Because our patient was examined 2 months after the exposure, options for making a diagnosis were limited. Extensive serologic cross-reactivity exists among the rickettsial species, which precludes the determination of species in our case. Although antibodies to rickettsiae can be long-lived, the extremely high levels of immunoglobulin (Ig) G and IgM suggest a recent rickettsial infection in our patient. Testing was unavailable for other rickettsiae (e.g., *R. parkeri*, *R. massiliae*).

Our patient likely had rickettsial infection acquired in Honduras. We present this case to alert clinicians to consider the diagnosis of rickettsial infections in the Americas, even if infections have not been previously documented in a specific country or region. Because rickettsial infections can be severe and are treatable, the clinician should consider rickettsial infections in returned travelers with compatible clinical findings. Our case also demonstrates the potential role of travelers as sentinels of emerging infectious diseases.
